# Lipid-derived reactive aldehydes link oxidative stress to cell senescence

**DOI:** 10.1038/cddis.2016.275

**Published:** 2016-09-08

**Authors:** Amy C Flor, Stephen J Kron

**Affiliations:** 1 Ludwig Center for Metastasis Research, Department of Molecular Genetics and Cell Biology, The University of Chicago, Chicago, IL 60637, USA

Genotoxic stress can induce proliferative cells to undergo accelerated senescence, where they remain viable but enter an irreversible cell cycle arrest similar to that of replicative senescence, including characteristic changes in gene expression, metabolism, and cell morphology.^1^ Even though transformed and tumor cell lines are considered immortal, they can be induced to undergo accelerated senescence. This extends to tumors *in vivo*, where ionizing radiation or chemotherapy promotes therapy-induced senescence (TIS). Although it remains controversial whether cancer cell senescence is a desirable outcome of cancer treatment, there is limited evidence to suggest that senescent cells in tumors may have beneficial effects, potentially including stimulation of antitumor immunity.

Whereas replicative senescence is linked to telomere shortening and the resulting DNA damage signal, accelerated senescence is associated with diverse stimuli, including replication stress, chromosomal damage, oxidative stress, mitogenic signaling, chromatin disruption, mitochondrial dysfunction, metabolic deregulation, and oncogene activation. For over a decade, prevailing opinion has remained that senescence inducers converge on DNA damage response pathway activation, which leads to the cell cycle arrest and downstream signaling that determine the senescent cell phenotype.^2^ In particular, oxidative stress has been linked to DNA damage at telomeres, accelerating their shortening and precipitating replicative senescence.^3^ Although the DNA damage response model for senescence has been useful, there has never been a direct test of whether DNA damage is both necessary and sufficient to induce senescence. Might other factors be critical?

A major limitation to progress in the field has been a lack of reliable cellular reporters for senescence, particularly for living cells. Most studies continue to rely on the classical senescence-associated *β*-galactosidase (SA-*β*-Gal) assay in fixed and permeabilized cells, detecting cleavage of the chromogenic substrate X-Gal (5-bromo-4-chloro-3-indolyl-*β*-D-galactopyranoside) to form a blue precipitate. Cell-permeable, fluorogenic *β*-galactosidase substrates offer an attractive alternative for live-cell studies. The fluorescein galactoside C_12_-FDG (5-dodecanoylaminofluorescein di-*β*-D-galactopyranoside) can detect SA-*β*-Gal in living cells, but fluorescein (*λ*_ex_/*λ*_em_, 490/514 nm) spectrally overlaps autofluorescence produced by the abundant age-related pigment lipofuscin,^4^ confounding the specificity of the detected signal.

As described in our recent report in *Cell Death Discovery*,^5^ we developed a new strategy for detection of SA-*β*-Gal in living senescent cells using the near-infrared *β*-galactosidase reporter DDAOG (9H-(1,3-dichloro-9,9-dimethylacridin-2-one-7-yl)-*β*-D-galactopyranoside).^6^ Although DDAOG is itself a fluorescent (460/610 nm), enzymatic cleavage to form DDAO (645/660 nm) shifts excitation 200 nm and emission 50 nm. Importantly, using DDAOG instead of C_12_-FDG enables simultaneous detection of SA-*β*-Gal by near-infrared fluorescence and lipofuscin by its green autofluorescence, providing a robust signature for cellular senescence (Figure 1a).

Advances in technology and fluorescent probes have helped make flow cytometry a robust tool for high-content screening of the effects of small molecules on complex cellular phenotypes. Toward establishing a new tool for senescence screening, we developed a dual parameter flow cytometry assay for SA-*β*-Gal and lipofuscin that readily detected induction of senescence in murine melanoma tumor cells after treatment with the DNA topoisomerase II poison etoposide. Testing a panel of topoisomerase inhibitors yielded multiple hits, including several more effective than etoposide. A single 25 Gy dose of gamma irradiation also strongly induced senescence. Toward validating the flow cytometry senescence assay as a tool for screening, we applied the assay to screen tumor cells treated with 36 redox-active small molecules along with a 5 Gy senescence-sensitizing dose. This identified four compounds that induced accelerated senescence only in combination with radiation.

In searching for a form of oxidative stress shared among conditions that strongly induced accelerated senescence, we observed a common pattern of increased lipid peroxidation and a resulting accumulation of lipid-derived aldehydes such as 4-hydroxy-2-nonenal (4-HNE, Figure 1b). Whereas treating cells with 4-HNE or 5 Gy alone yielded only low levels of senescence, combining 4-HNE and 5 Gy could recapitulate the effects of etoposide. Further implicating lipid peroxidation in accelerated senescence, the aldehyde sequestering drug hydralazine^7^ blocked senescence induction by 25 Gy, etoposide, and the other topoisomerase inhibitors. Our interpretation of these results is that radiation and chemotherapy must not only produce DNA damage but also cause lipid peroxidation to drive TIS.

Lipid peroxidation and its end-products, the lipid-derived aldehydes such as 4-HNE, have long been considered to have important roles in redox signaling, oxidative stress, and cellular and organismal aging.^8, 9^ Suggesting direct effects, bystander senescence in proliferating cells growing near senescent cells in tissue culture or in tumors has been linked to exposure to 4-HNE released by the senescent cells.^10^ Lipid peroxidation is a form of oxidative damage induced by hydroxyl radicals, hydrogen peroxide, or other reactive oxygen species (ROS) reacting with the polyunsaturated fatty acids in cell membranes, organelles, and fat droplets. The resulting lipid hydroperoxides initiate a free radical cascade terminating in reactive aldehyde end-products such as 4-HNE, malondialdehyde, and acrolein. Electrophiles such as 4-HNE can undergo Michael addition reactions with cellular nucleophiles, including glutathione and cysteine thiols, lysine *ɛ* amino groups, and histidine imidazoles, leading to protein carbonylation. Adducts can initiate Schiff base formation, producing intra- and inter-protein cross-links. For example, the age-related autofluorescent pigment lipofuscin is formed by intracellular accumulation and aggregation of aldehyde adducts.^11^

Although high levels of lipid peroxidation are toxic, likely via inducing DNA damage and targeting mitochondria, lower levels are well tolerated due to physiological activation of the Keap1/Nrf2 antioxidant response pathway. Lipid aldehydes are detected by highly reactive cysteine thiols on the Keap1 sensor protein.^12^ Keap1 carbonylation releases the transcription factor Nrf2 to bind antioxidant response element sites and upregulate antioxidant and detoxification genes such as glutathione reductases, GST, NQO1, ALDH, AKR, HO-1, thioredoxin, ferritin, and others. Thus, 4-HNE can serve as a second messenger to induce an adaptive response, increasing overall tolerance for oxidative stress and resisting cell death.

Given the complex biology, how aldehyde end-products of lipid peroxidation might cooperate with DNA damage to promote cellular senescence remains unclear. Accumulation of lipofuscin may be more than simply a marker of senescence,^13^ but signaling by lipid peroxidation-derived aldehydes likely serves a key role. Examining Keap1 as a 4-HNE target appears particularly promising, given the known interactions between Nrf2 and senescence-related factors p21^14^ and caveolin.^15^

Taken together, the data generated by this study argue that lipid peroxidation to produce reactive lipid species such as 4-HNE cooperates with DNA damage to drive tumor cells toward TIS (Figure 1c). Although further investigation of downstream signaling from 4-HNE and its interactions with DNA damage responses will be necessary, this work may lead to a new understanding of TIS *in vivo* and provide opportunities to improve patient responses to conventional and targeted cancer therapies.

## Figures and Tables

**Figure 1:**
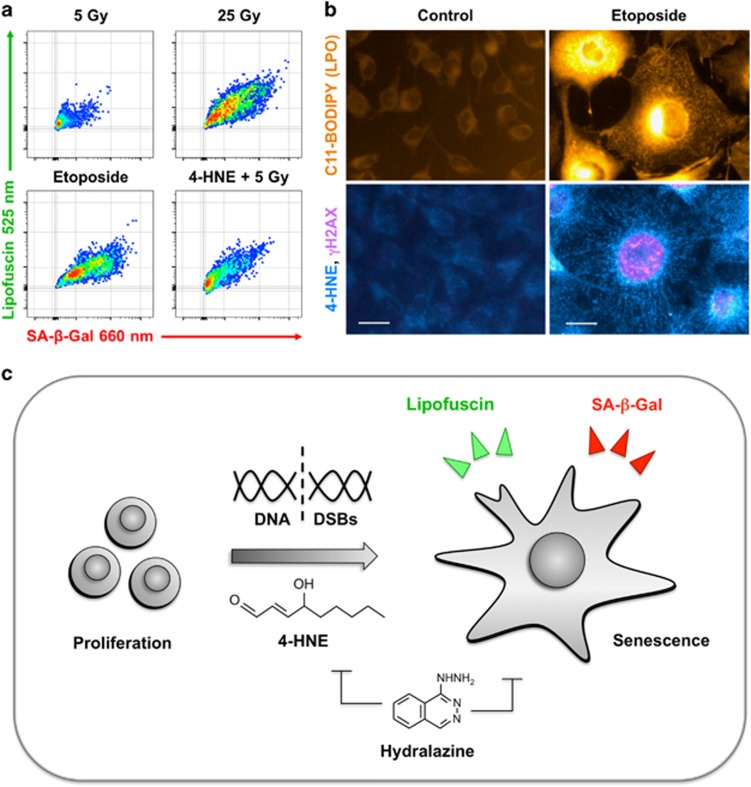
The aldehyde end-product of lipid peroxidation 4-HNE synergizes with DNA damage to induce accelerated senescence. (**a**) Detection of accelerated senescence in live cells by dual parameter flow cytometry using the far-red fluorescent probe DDAOG to measure senescence-associated beta-galactosidase (SA-*β*-Gal) and green autofluorescence to detect the age-related pigment lipofuscin. Treating B16 murine melanoma cells with 5 Gy of gamma irradiation shifts only a small fraction into senescence compared with a higher dose of 25 Gy or the topoisomerase II poison etoposide. Combined treatment with 4-HNE and 5 Gy drives more cells into senescence, demonstrating a compound effect. (**b**) Inducing senescence in melanoma cells with etoposide results in high levels of lipid peroxidation, 4-HNE adducts, and chromosomal breaks compared with untreated controls. Upper micrographs show lipid peroxidation reporter C11-BODIPY (LPO, orange). Lower images display dual immunofluorescence staining for 4-HNE adducts (cyan) and the DNA double-strand break (DSB) marker pH2AX (magenta). Bar=5 *μ*m. (**c**) Schematic of our findings. We observed onset of accelerated senescence with a flow cytometry assay for SA-*β*-Gal and lipofuscin when proliferating cancer cells were treated with a high dose of radiation or topoisomerase poisons. Along with DNA DSBs, radiation and topoisomerase inhibitors each induced lipid peroxidation, leading to accumulation of reactive aldehydes such as 4-HNE. Treating cells with the aldehyde scavenging compound hydralazine blocked the effects of radiation or topoisomerase inhibitors, establishing a key role for lipid peroxidation in accelerated senescence
